# Application of multi-layer denoising based on ensemble empirical mode decomposition in extraction of fault feature of rotating machinery

**DOI:** 10.1371/journal.pone.0254747

**Published:** 2021-07-19

**Authors:** Kangping Gao, Xinxin Xu, Jiabo Li, Shengjie Jiao, Ning Shi

**Affiliations:** 1 National Engineering Laboratory for Highway Maintenance Equipment, Chang’an University, Xi’an, China; 2 Henan Gaoyuan Maintenance Technology of Highway Co., Ltd., Xinxiang, China; US Department of Agriculture, UNITED STATES

## Abstract

Aiming at the problem that the weak features of non-stationary vibration signals are difficult to extract under strong background noise, a multi-layer noise reduction method based on ensemble empirical mode decomposition (EEMD) is proposed. First, the original vibration signal is decomposed by EEMD, and the main intrinsic modal components (IMF) are selected using comprehensive evaluation indicators; the second layer of filtering uses wavelet threshold denoising (WTD) to process the main IMF components. Finally, the virtual noise channel is introduced, and FastICA is used to de-noise and unmix the IMF components processed by the WTD. Next, perform spectral analysis on the separated useful signals to highlight the fault frequency. The feasibility of the proposed method is verified by simulation, and it is applied to the extraction of weak signals of faulty bearings and worn polycrystalline diamond compact bits. The analysis of vibration signals shows that this method can efficiently extract weak fault characteristic information of rotating machinery.

## Introduction

Identification and elimination of fault conditions in rotating machinery significantly increase the working efficiency and service life. Since the failure of rotating machinery can cause accidental damage to the equipment, to ensure the long-term and safe operation of the equipment, timely monitoring of the early operating status of the rotating machinery is essential. However, considering the complex and changeable physical signal transmission path, environmental interference, and human factors, the signal contains considerable noise, and it is difficult to extract the characteristic data of the device. Therefore, denoising the early weak signals of rotating machinery and extracting fault features have become the research topic of many scholars [1–4]. Due to the high sensitivity of vibration signals to the failure degree of rotating machinery, extracting the fault characteristics of rotating machinery from the vibration signals has become a key link in condition monitoring. The feature extraction can be carried out in the time domain, frequency domain, and time-frequency domain [5–7]. When rotating machinery has a local failure, pulses will be periodically generated during the working process. At this time, the collected vibration signal has the characteristics of non-stationary and non-linearity, which makes it difficult to extract mechanical failure features.

Various signal processing methods have been applied to solve this feature extraction problem, including short-time Fourier transform (STFT) [8, 9], Wavelet transform (WT) [10–14], Sparse decomposition (SD) [15], etc. Although these methods can reduce the interference of noise on weak signals, they all have their shortcomings. For example, WT needs to determine the wavelet base and mother wavelet in advance. Also, although SD has good signal decomposition capabilities, it has a strong dependence on the aton library and decomposition methods. Therefore, the above methods are not suitable for processing nonlinear and non-stationary vibration signals containing a lot of noise. For this reason, some scholars have proposed time-frequency domain adaptive analysis methods to analyze vibration signals, such as empirical mode decomposition (EMD) and local mean value decomposition (LMD) [16–21]. EMD can decompose a complex signal into a limited number of intrinsic modal function (IMF) components and is an efficient method for decomposing signals. However, when the information in a certain frequency band of the signal to be measured is not continuous, modal aliasing and end effect will occur when the signal is decomposed by EMD, which makes the IMF component lose its physical meaning.

To avoid the occurrence of end effect and modal aliasing, many scholars have proposed the ensemble empirical mode decomposition (EEMD) method [22–24] when extracting fault features. By changing the characteristics of the extreme points of the fault signal, the modal aliasing and end effects caused by abnormal disturbances are effectively suppressed. Among them, Li et al. [22] combined improved frequency band entropy and EEMD for fault diagnosis of rotating machinery and improved ability to remove noise in the process of fault feature extraction. Žvokelj et al. [23] combine independent component analysis (ICA) with the EEMD method, adaptively decomposes the signal into different time scales, and realizes the fault diagnosis of the bearing while providing a multivariable signal denoising mechanism. Guo et al. [24] use the combination of EEMD, WT and the modulation signal bispectrum to perform multi-level noise reduction of signal characteristics. The analysis results show that this method is more accurate and reliable in diagnosing bearing faults than using EEMD alone.

Although the above method solves the problems of mode aliasing and end effect, considerable noise still remains in the decomposed IMF component. Therefore, the problem of denoising IMF components has become the core of dynamic monitoring and fault diagnosis of rotating machinery. Among them, some multi-scale noise reduction analysis methods have received widespread attention. Liu et al. [25] combined LMD and wavelet noise reduction to perform fault diagnosis of large power equipment. Wang et al. [26] used the energy method to eliminate and update the PF component obtained from the EMD decomposition and used the envelope spectrum entropy to determine the optimal modal component and penalty factor of the variational modal decomposition. By combining the two to extract the fault features of the gearbox, the method is demonstrated to be superior to EMD for fault diagnosis. In recent years, some scholars have combined the multi-scale analysis method with the singular value decomposition (SVD) method to improve the efficiency of noise reduction. For example, the combination of the SVD and Hankel matrix-based denoising method in Ref [27] improves the reliability of bearing fault detection. Tian et al. [28] combined the LMD-SVD with the extreme learning machine for bearing fault diagnosis, which shortened the time for fault identification. Jiang et al. [29] combined the adaptive Morlet wavelet and SVD to de-noise the vibration signal. However, there are some shortcomings in the above-combined methods, which may cause some important information to be lost; among them, SVD is not very effective for denoising small pulses in vibration signals. WTD can reduce the interference of noise on characteristic signals, but it cannot extract characteristic signals efficiently [30].

ICA is a method of redundancy cancellation based on high-order statistical analysis theory. When the source signal is unknown, independent source signals are reconstructed from multiple observed mixed signals and are not affected by interference noise. It is suitable for the detection and extraction of early weak signals. Considering the unique advantages of ICA noise reduction, it is widely used in many fields such as medical signal processing [31, 32] and rotating machinery dynamic monitoring [33, 34]. However, ICA requires that the number of observation signals is greater than or equal to the number of signal sources when extracting weak signals, which is not applicable in the common single-channel underdetermined situation.

Based on the above problems, and considering the weak early fault signals of the equipment, variable working conditions, and the presence of external environmental interference, a multi-stage noise reduction method based on EEMD to realize the feature extraction of weak signals is proposed, which overcomes the defect that a single method is difficult to achieve high-precision fault detection. The original signal of the faulty equipment is decomposed by the principle of EEMD, and the single-channel vibration measurement data is decomposed into a series of IMF components. Since the original signal contains noise, after EEMD decomposition, the generated IMF component is composed of a true component and a pseudo component. Therefore, to highlight the characteristics of the fault, the IMF components are processed by a multi-layer noise reduction method: the first layer of noise reduction uses a multi-index comprehensive evaluation method to select IMF components that contain more original signals; the second layer of noise reduction uses WTD to process the selected IMF components. Also, the IMF components processed by WTD are used as observation signals. By constructing a virtual noise channel, using the FastICA algorithm to unmix and separate it, the useful signal and the noise signal are obtained, and the envelope spectrum of the useful signal is analyzed to highlight the characteristic frequency. The framework of the rest of this article is as follows: Section 2 introduces the basic principles of EEMD. Section 3 analyzes the multi-layer noise reduction algorithm. Section 4 uses simulation signals to verify the feasibility of the proposed method. Section 5 applies the proposed method to the extraction of weak features of faulty bearings and worn polycrystalline diamond composite (PDC) bits. The article concludes in the last Section.

## The basic principle of EEMD

The core of the EEMD method is EMD. Although EMD is efficient when decomposing the signal, if the signal in a certain frequency band of the signal to be measured is not continuous, it will cause modal aliasing in the decomposed signal. To solve this problem, white noise is usually added to the original signal. Also, considering the feature that the mean value of white noise is zero, the lumped average calculation is performed on the effect after multiple decompositions, which avoids the interference of the introduced white noise on the fault feature extraction. The process of EEMD decomposition is as follows:

1): To construct the original signal *x*_*i*_(*t*), add N-th Gaussian white noise *n*_*i*_(*t*) with zero mean and constant standard deviation to the input signal *x*(*t*). The equation is as follows:


$$x_{i}(t)=x(t)+n_{i}(t)$$ (1)

Among them, *i* = 1,2,…,*N*, *N* is generally 100–300. After research [35], the added Gaussian white noise conforms to the following law:

$$\varepsilon =\frac{k}{N}$$ (2)

The formula *ε* represents the standard deviation of the signal and *k* represents the intensity of added noise.

2): Use the EEMD method to decompose the signal *x*_*i*_(*t*) into a series of IMF components and margins, and get the following equation:


$$x_{i}(t)=\sum_{j=1}^{m}c_{i,j}(t)+r_{iN}(t)$$ (3)

Among them, m is the number of IMF components, *c*_*i*,*j*_(*t*) is the *j*-th IMF component of the *i*-th experiment, *i* =1,2,…,*N*; *j* = 1,2,…,*m*.

3): Because in each decomposition step, the sequence of noise is different, and there is no correlation between them. Therefore, to offset the influence of noise, sufficient experimental steps are taken, and the aggregate mean value of the corresponding IMF components is taken as the final result:


$$c_{j}(t)=\frac{1}{N}\sum_{i=1}^{N}c_{i,j}(t)$$ (4)

Where *c*_*j*_(*t*) indicates the *j*-th IMF decomposed by EEMD.

It can be seen that the EEMD method can enhance the modulation component caused by the failure of the rotating machinery, thereby suppressing the noise component.

## Multi-layer noise reduction method

**Fig 1:**
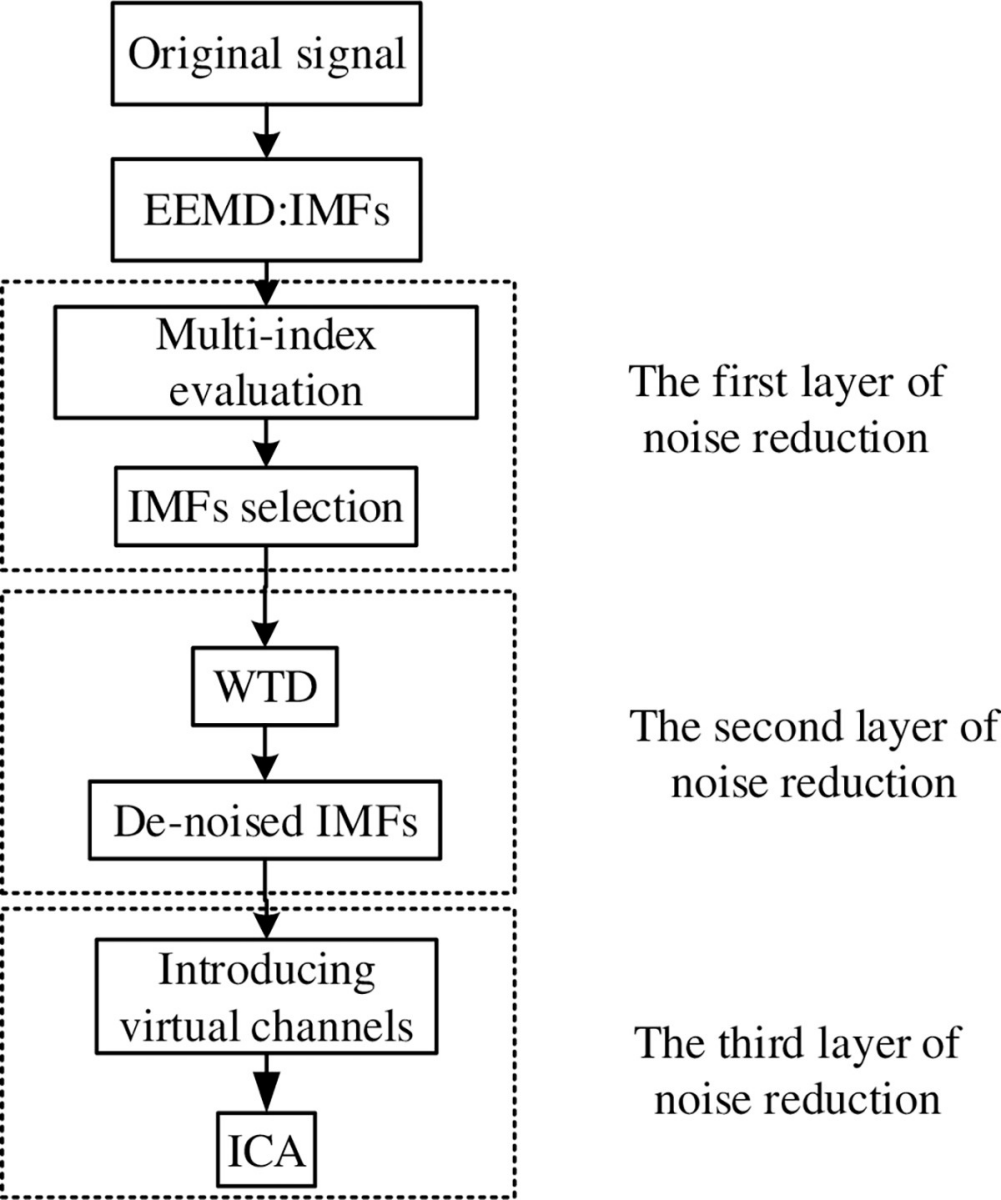
The multilayer noise reduction algorithm scheme.

The early fault signal strength of rotating machinery is weak, contains a lot of noise, and is non-linear and non-stationary, which makes it difficult to extract fault features. Therefore, to avoid the interference of external noise on the weak signal, this article has carried out multi-layer noise reduction processing on the original vibration signal, and the flowchart is shown in Fig 1. The vibration signal of the faulty equipment is decomposed by EEMD to obtain a series of IMF components, it shows the local signal of the fault. However, this signal contains a lot of interference noise (e.g., Gaussian white noise, impact noise, interference noise between devices), which is not conducive to highlighting the fault frequency. Therefore, use the comprehensive evaluation index to select the IMF components that contain more original information, and perform WTD on the selected IMF components. Also, the remaining components are summed to construct a virtual channel. Then use the FastICA algorithm to reduce noise and unmix to separate the noise from the useful signal. The multi-layer noise reduction method can eliminate powerful noise interference, when feature extraction is performed on the fault signal, the characteristic frequency extraction performance is enhanced, which is beneficial to highlight the fault characteristics.

### Principle of IMF selection

Considering that the IMF component is not entirely a useful signal, to improve the fault detection performance, the IMF component containing more original signals is extracted. Among them, the correlation coefficient (CC) [36] reflects the degree of similarity between the original signal and the output signal. The higher the CC value, the greater the similarity between the input signal and the output signal. Therefore, CC is used as an index for selecting IMF components. CC is defined as follows:

$$\mathrm{CC}=\frac{\sum_{i=1}^{N}\left(y_{i}-\bar{y})(x_{i}-\bar{x}\right)}{\sqrt{\sum_{i=1}^{N}{\left(y_{i}-\bar{y}\right)}^{2}}\sqrt{\sum_{i=1}^{N}{\left(x_{i}-\bar{x}\right)}^{2}}}$$ (5)

Among them, $\bar{y}$ and $\bar{x}$ are the average values of the output signal *y*(*t*) and the input signal *x*(*t*), respectively.

Although CC has a certain sensitivity to noise, to avoid the loss of characteristic information, some other methods (kurtosis [37], root mean square (RMS) [38]) are also used as the selection index of IMF components. Kurtosis is a numerical statistic that reflects the distribution of signal amplitude, and it has a certain sensitivity to vibration and shock signals, which is highly efficient in describing the pulse characteristics of vibration signals. The model is as follows:

$${\mathrm{Kurtosis}}_{x}=\frac{M\sum_{a=1}^{M}{\left(x_{i}-\bar{x}\right)}^{4}}{{\left(\sum_{a=1}^{M}{\left(x_{i}-\bar{x}\right)}^{2}\right)}^{2}}$$ (6)

Among them, $\bar{x}$ is the mean value of signal x, and M is the number of sampling points.

The RMS is defined as follows:

$${\mathrm{RMS}}_{x}=\sqrt{\frac{\sum_{a=1}^{M}x_{i}{}^{2}}{M}}$$ (7)

Among them, M is the number of sampling points.

Because the early fault signals of rotating machinery are weak and contain a lot of noise, CC only considers the similarity between the input signal and the output signal. RMS alone is not efficient for the early feature extraction of weak signals. Although the kurtosis is highly sensitive to impact signals, when the fault is severe, the kurtosis cannot maintain a rising trend during the full life cycle of the rotating machinery, and it is only efficient in the early fault identification of the rotating equipment. Therefore, considering the shortcomings of a single index when selecting IMF components, this paper proposes a comprehensive evaluation index to select IMF components, which avoids wrong signal selection in the process of single index evaluation. The EEMD principle is used to decompose the original signal into a series of IMF components, and the CC, kurtosis, RMS, energy ratio (the ratio of the IMF component to the original signal) of the i-th component [39] is defined as H_i,1_,H_i,2_,H_i,3_,H_i,4_. In the process of feature extraction of rotating machinery, due to the complex working environment, different indicators have different diagnostic capabilities for different fault degrees. Therefore, each indicator is given the same weight, and a comprehensive evaluation indicator *Q*_*i*_ (the average of the four evaluation indicators) is defined. This method avoids the shortcomings of loss of characteristic information when selecting IMF for a single indicator. Set the threshold for the comprehensive performance evaluation index: To retain most of the important information, select the IMF component to occupy more than 85% of the original information.

### IMFs denoising based on wavelet threshold

After EEMD decomposition of the original signal, a series of IMF components are obtained, all of which do not completely contain the real information of the vibration signal. Although the IMF components containing the main information were selected by using the comprehensive evaluation index, these components were still covered by a lot of noise. To avoid noise interference, further de-noising of decomposed IMF components has become the key to improve the fault diagnosis of rotating machinery.

WT is widely used in the field of signal noise reduction, using multi-resolution time-frequency distribution diagrams to represent the signal and decomposing the signal into different sizes. WTD is a WT-based noise reduction algorithm. Discrete Wavelet Transform (DWT) is described as follows:

$$WT(a,b)=\frac{1}{\sqrt{2^{a}}}\sum_{t=1}^{T}s(t)\times \psi (\frac{t-b\cdot 2^{a}}{2^{a}})$$ (8)

Among them, a is the decomposition level, b is the translation parameter, T is the number of sampling points, and *ψ* is the wavelet basis function.

Performing WTD on the main IMF components, to some extent, avoids the shortcomings of WTD directly acting on the original signal and causing some useful signal loss. The basic principle of wavelet threshold is to use high-amplitude components to reconstruct the denoised information, and wavelets smaller than the threshold are set to zero. Choosing an appropriate threshold method is very important for WTD. Two main threshold operators [34]: hard threshold and soft threshold are described as Eqs (9) and (10), respectively.


$${\overset{⏜}{\omega }}_{j,k}=\{\begin{array}{l} \omega_{j,k}\;|\omega_{j,k}|\geq \lambda \\ 0\;|\omega_{j,k}|<\lambda \end{array}$$ (9)

$${\overset{⏜}{\omega }}_{j,k}=\{\begin{array}{l} \mathrm{sgn}(\omega_{j,k})(\omega_{j,k}-\lambda )\;|\omega_{j,k}|\geq \lambda \\ 0\;|\omega_{j,k}|<\lambda \end{array}$$ (10)

Among them, *ω*_*j*,*k*_ is the k-th wavelet coefficient of the j-th layer of the wavelet transform, and *λ* is the threshold.

Compared with the hard threshold method, the reconstruction coefficient of the soft threshold method has good continuity at the threshold point, and there is no breakpoint. Therefore, this article chooses the soft threshold method to apply to WTD.

### ICA extracts characteristic components

WTD has good noise reduction performance, but it is not very effective for the extraction of weak signals. Considering that ICA is a blind source separation technology when the source signal is unknown, the source signal is reconstructed from the observed mixed signal, and it is unmixed and denoised to highlight the weak characteristics. Because of its unique advantages, it is widely used in the extraction of weak signals. Taking into account the underdetermined single channel in the project, the IMF component processed by WTD is used as the observation signal, and the rest of the IMF components are summed and reconstructed into the virtual noise channel signal. The observation signal and the virtual noise channel signal are used as the input matrix of FastICA, which effectively solves the problem of underdetermined blind source separation.

Use FastICA to separate noise and useful source signals. The ICA model is described as follows:

X=AS (11)

Among them, *X*(*t*) = [*x*_1_(*t*),*x*_2_(*t*),…,*x*_*n*_(*t*)] is an n-dimensional observation signal, *s*(*t*) = [*s*_1_(*t*),*s*_2_(*t*),…,*s*_*m*_(*t*)] is an m-dimensional mutually independent source signal, and A represents an m×n unknown mixing coefficient matrix, and the number of observation points m is greater than or equal to the number of source signal points n.

The purpose of ICA analysis is to estimate the inverse matrix *A*^−1^ of the coefficient matrix A based on certain optimization criteria without prior knowledge. The independent signal source S can be obtained through the inverse matrix of A, that is *S* = *A*^−1^*X*, which is used to calculate the independent signal. Where the inverse matrix *A*^−1^ is represented by W, and the following expression is obtained:

y(t)=W⋅x(t)=W⋅A⋅s(t) (12)

*y*(*t*) = [*y*_1_(*t*),*y*_2_(*t*),…,*y*_*N*_(*t*)]^*T*^ is an approximate estimate of the independent source signal.

**Fig 2:**
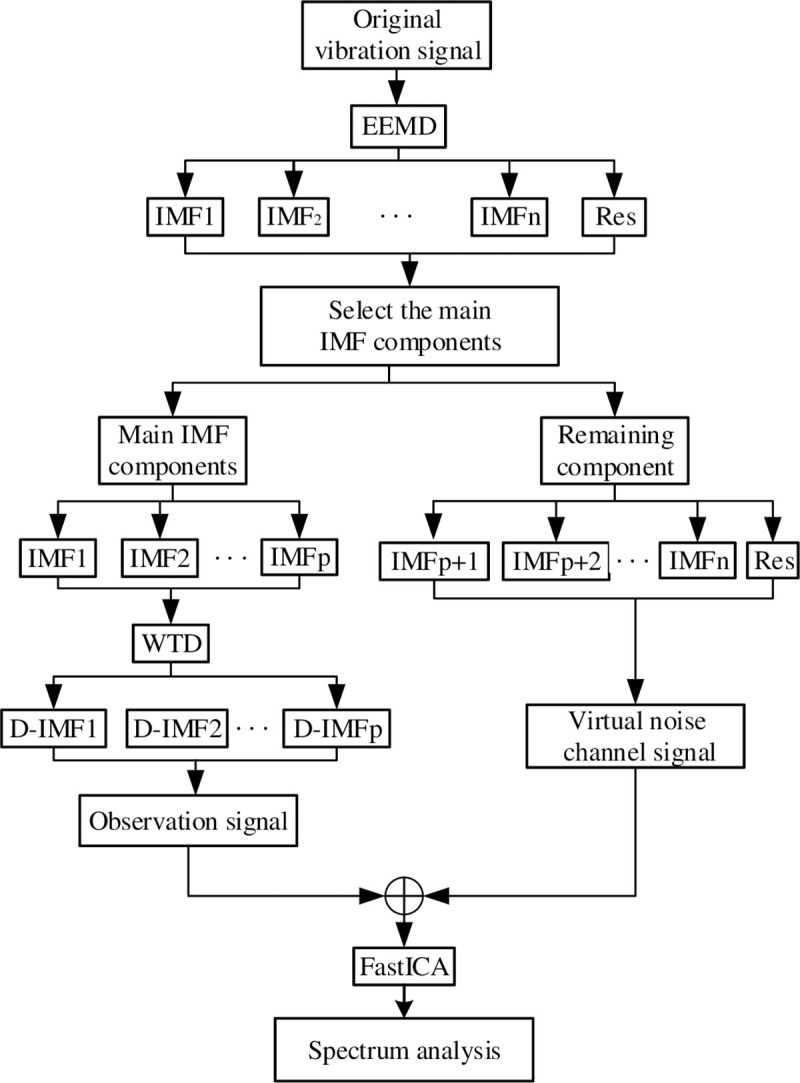
Multilayer noise reduction flow chart.

Finally, the flowchart of the fault extraction method for multi-layer noise reduction is shown in Fig 2. The specific implementation steps are as follows:

1. Decompose the vibration signal of the rotating machine through EEMD;
2. Calculate the CC, spectral kurtosis, RMS, and energy ratio of each IMF, find the *Q*_*i*_ value of each IMF component; also, select the main IMF component through the *Q*_*i*_ value;
3. Perform WTD noise reduction processing on the main IMF components;
4. Use the IMF component processed by WTD as an observation signal and introduce a virtual noise channel;
5. Use FastICA to unmix and separate the observed signal and virtual noise signal to obtain useful signals;
6. Perform spectrum analysis on useful signals to highlight characteristic signals.

## Simulation analysis

**Fig 3:**
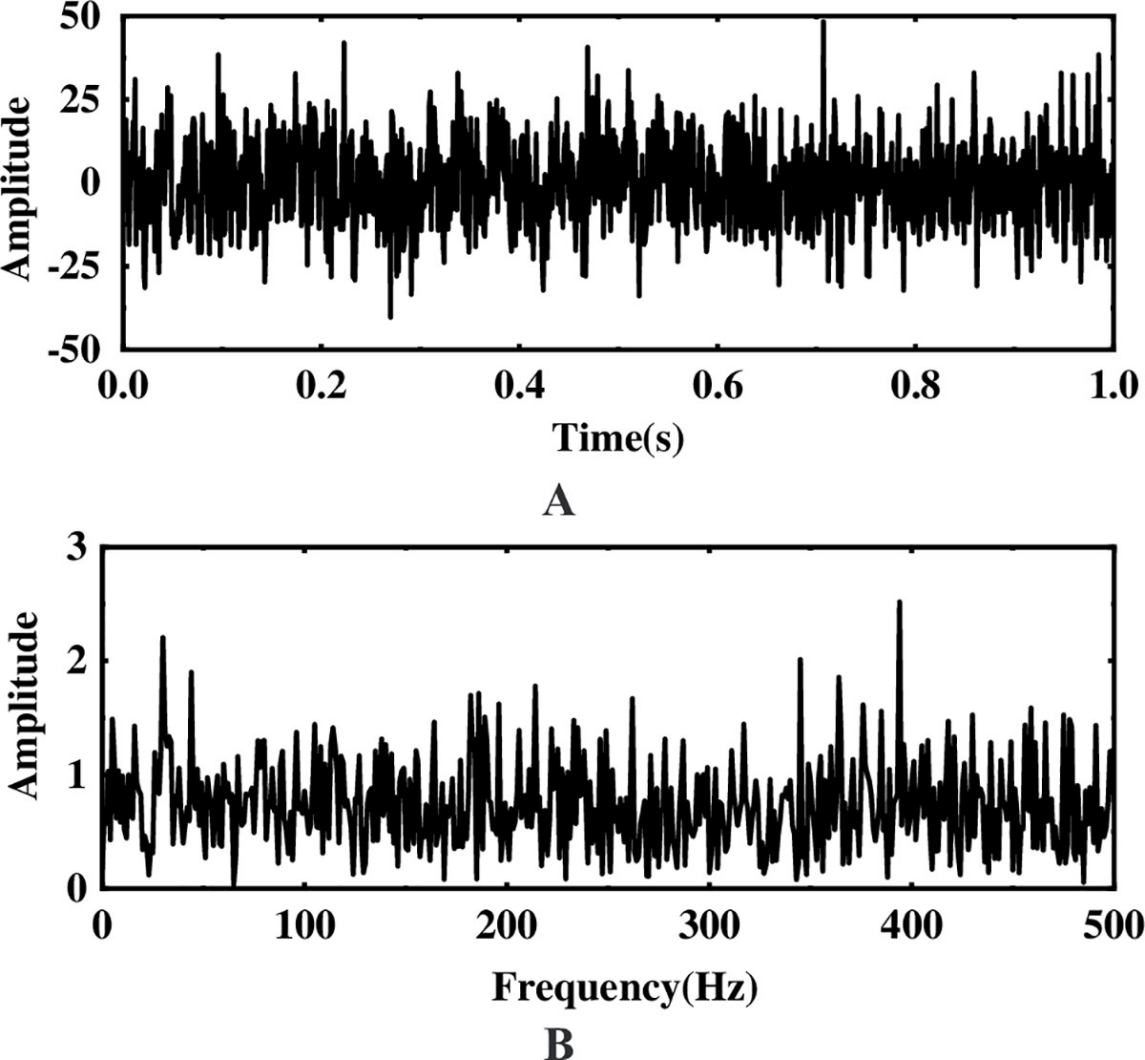
Original waveform and FFT spectrum of the simulated signal. (A) Original waveform (B) Spectrogram.

To verify the feasibility of the method proposed, a sine signal with Gaussian white noise is used for simulation analysis, where the simulated signal is *s* = 0.8sin(60*πt*), and the signal-to-noise ratio (SNR) with Gaussian white noise is -20dB. To avoid the occurrence of modal aliasing, when performing EEMD decomposition of the simulated signal, 100 times white noise with an amplitude of 0.2 times the standard deviation of the original signal is added. Fig 3 shows the time domain and frequency spectrum of the simulated signal, although there is a certain impact signal in the original waveform, the characteristic frequency of the signal is difficult to highlight in the frequency spectrum due to the interference of external noise.

**Fig 4:**
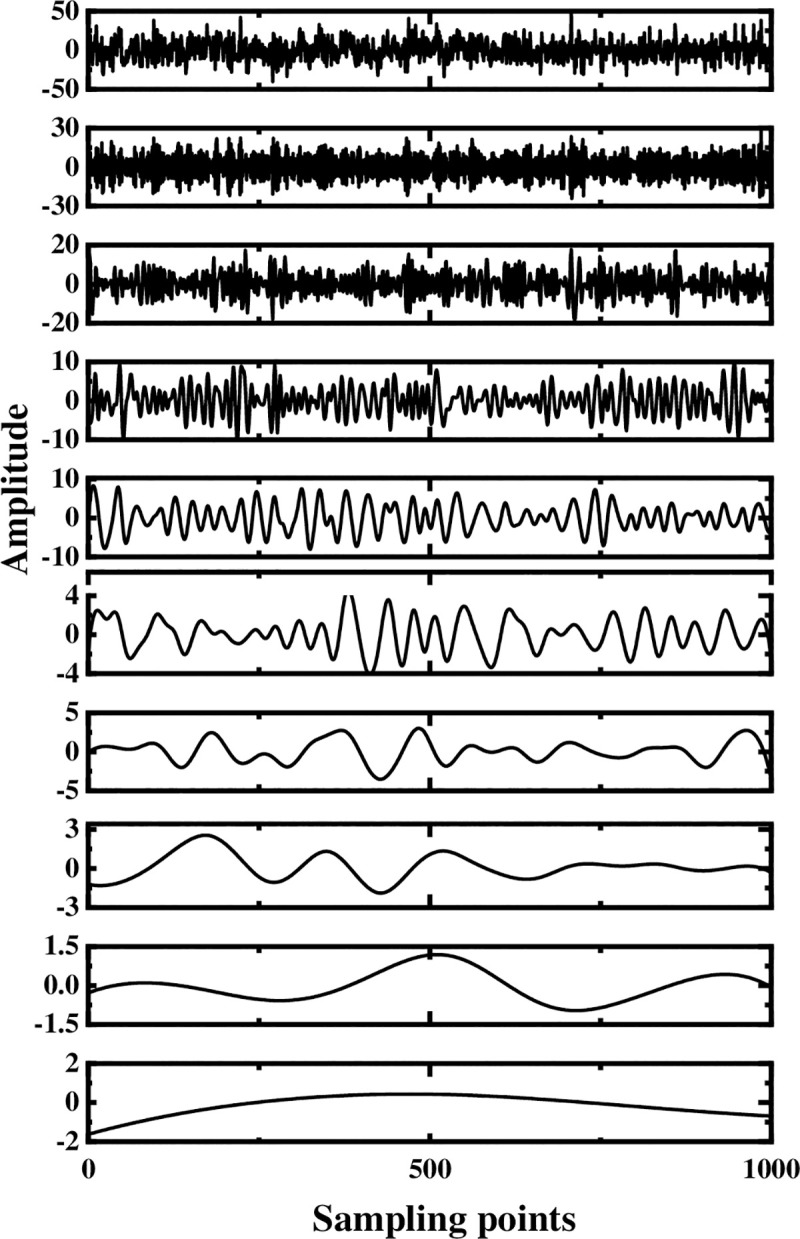
Decomposition result of original signal EEMD.

**Fig 5:**
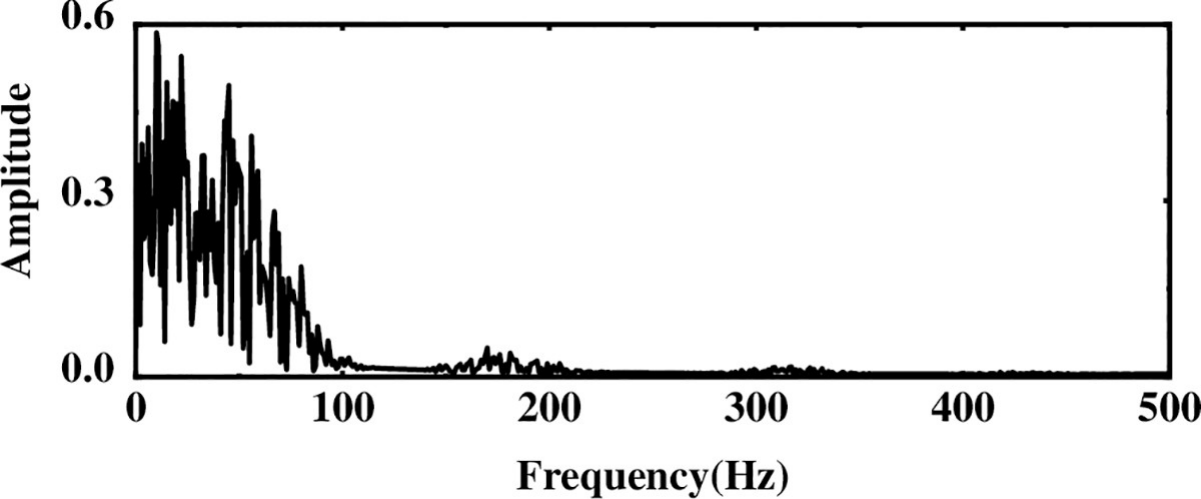
Spectrogram after WTD processing.

The multi-layer noise reduction method is applied to the simulation signal. Due to the interference of noise, each IMF component does not completely contain useful signals. Observing Fig 4, we can see that the impact signals of the first few-order IMF components are obvious. Considering the space, this section calculates the comprehensive evaluation indicators of the first six-order IMF components, as shown in Table 1. According to Table 1, the first fourth-order IMF components contain more than 85% of the original signal. Therefore, the first four-order IMF components are further processed by WTD, and the signal is reconstructed and analyzed by FFT after noise reduction. The result is shown in Fig 5. Comparing Figs 3 and 5, it is found that after WTD transformation, the noise is suppressed to a certain extent, but the characteristic signal is not prominent. Therefore, to highlight the characteristic frequency of the weak signal, further processing of the IMF component processed by the WTD is required.

**Table 1:** The *Q*_*i*_ value of the simulated signal IMF component.

	IMF_1_	IMF_2_	IMF_3_	IMF_4_	IMF_5_	IMF_6_
H_1_	0.531	0.216	0.125	0.062	0.021	0.009
H_2_	0.243	0.164	0.182	0.159	0.092	0.061
H_3_	0.569	0.157	0.093	0.068	0.035	0.027
H_4_	0.732	0.126	0.064	0.037	0.003	0.003
Q_i_	0.519	0.166	0.116	0.082	0.038	0.025

**Fig 6:**
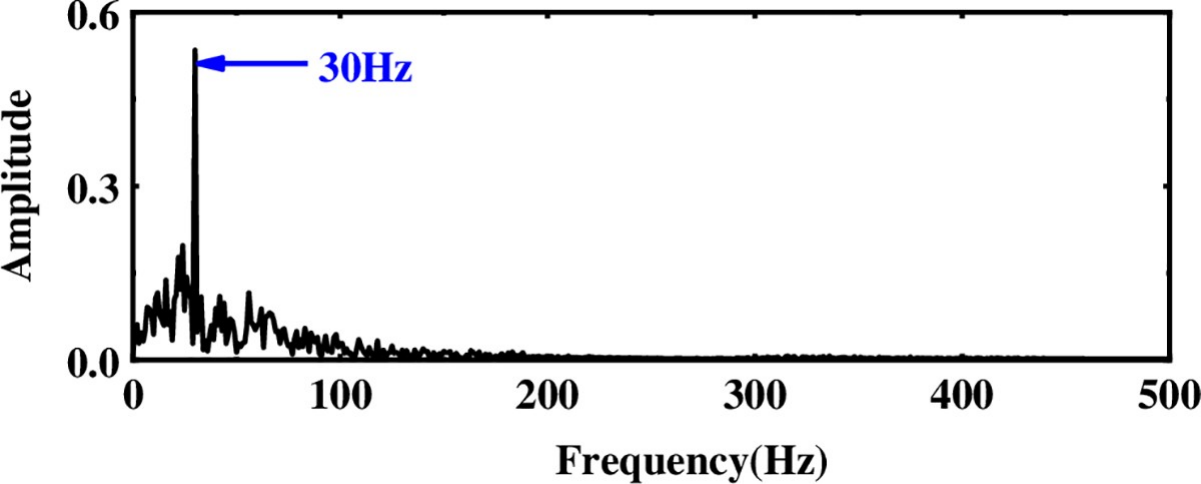
Spectrum after multi-layer noise reduction.

Next, to solve the problem of ICA single-channel underdetermination, this section uses the IMF component processed by WTD as the observation signal, and the sum of the remaining IMF components is used as the virtual noise channel signal. Introduce the virtual channel, use the observation signal and the virtual noise signal as the input matrix of ICA, and use the FastICA algorithm to reduce noise and unmix. The spectrum analysis of the unmixed useful signal is shown in Fig 6. Compared with the WTD noise reduction analysis, the noise interference is suppressed after the ICA processing, and the characteristic amplitude of the simulated signal is highlighted.

**Fig 7:**
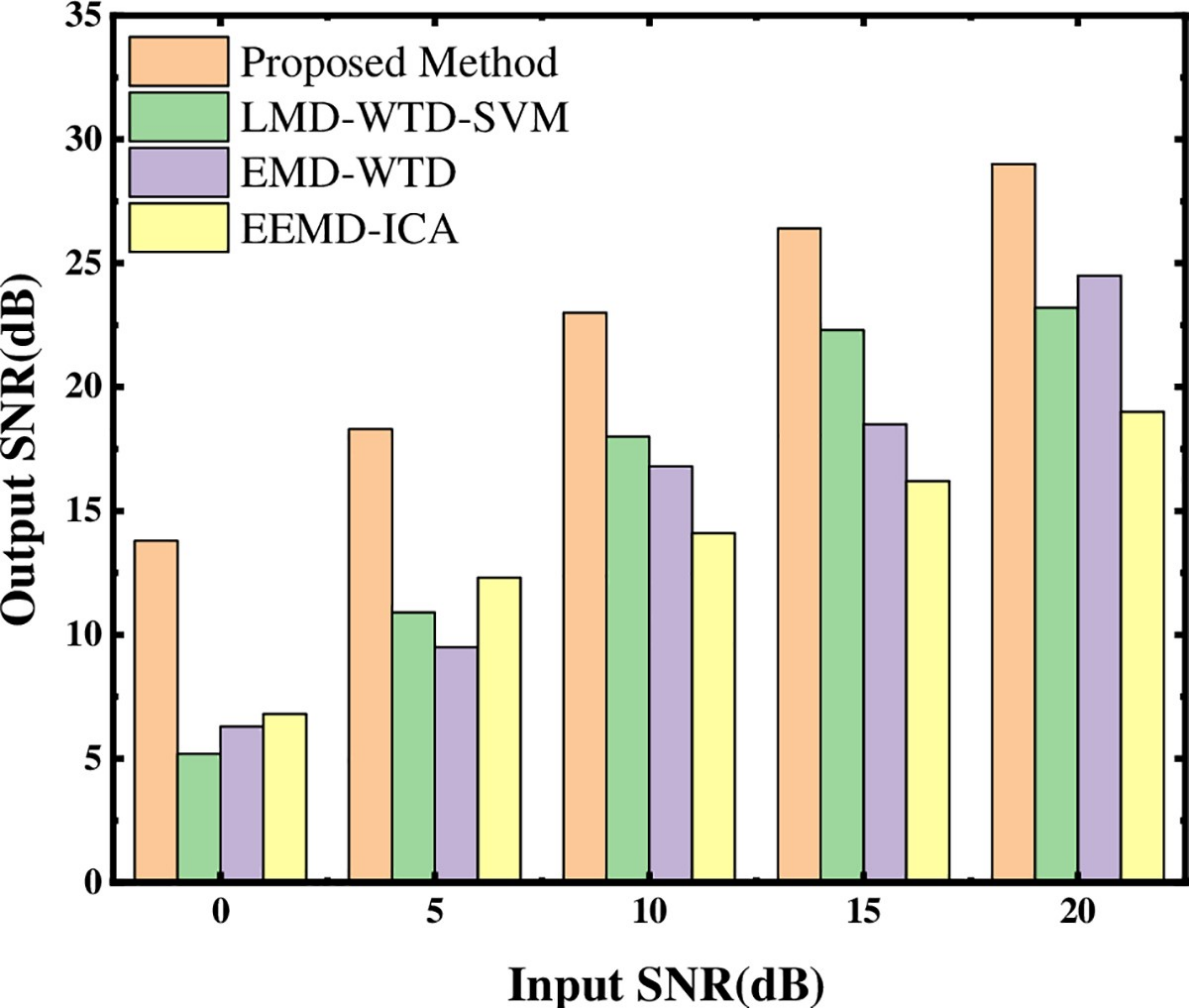
Noise reduction effect of four methods under different noise SNR.

To evaluate the advantages of the proposed method, three methods (EMD-WTD [40], EEMD-ICA [41], LMD-WTD-SVM [30]) were used to process the same signal and perform comparative analysis. Adding different degrees of Gaussian white noise (0, 5, 10, 15, and 20dB) to the sinusoidal signal, the evaluation index of noise reduction performance is the SNR. Under the same noise interference, the larger the SNR after noise reduction processing, the better the noise reduction performance of this method. Under different noise interference, the SNR processed by different noise reduction methods is shown in Fig 7. Comparing these four methods, it can be found that the noise reduction performance of the proposed method is significantly better than the other three methods, which again verifies the effectiveness of the proposed method.

## Experimental analysis and engineering application

### Failure bearing analysis

#### Data acquisition

**Fig 8:**
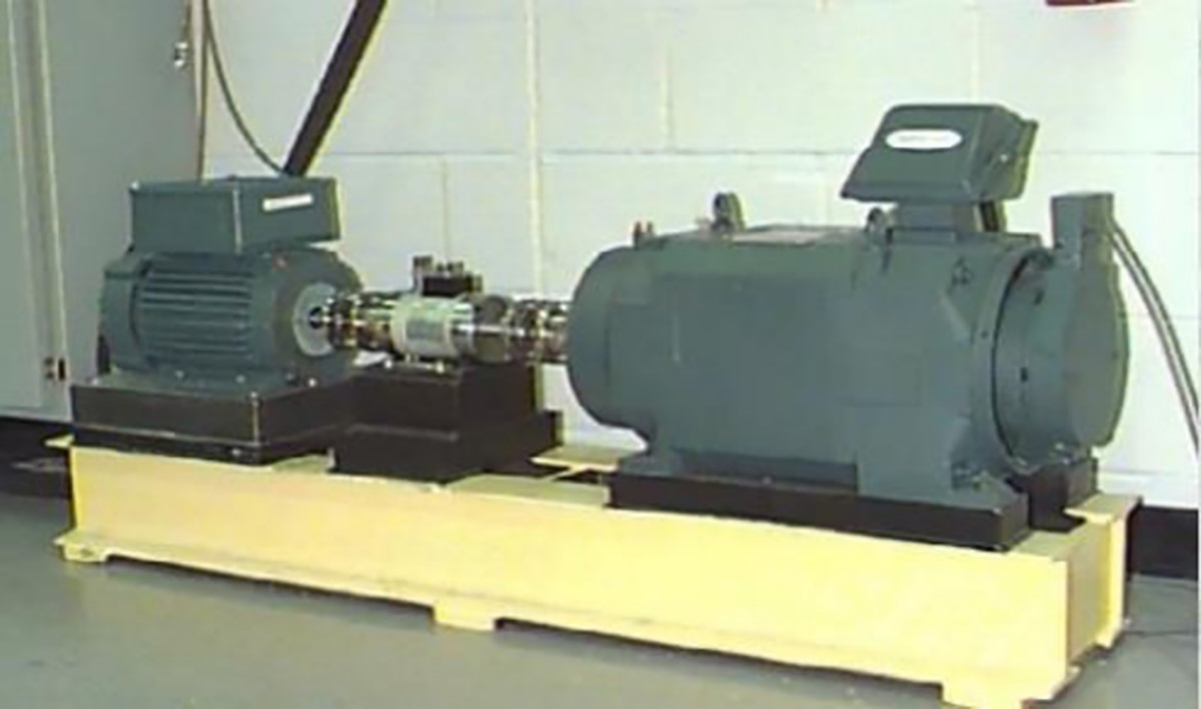
Experimental platform for failed bearings.

This section uses the data of the inner-race fault bearing of Western Reserve University [42] to conduct experiments to analyze and verify the effectiveness of the proposed multilayer noise reduction method. The experimental device is shown in Fig 8. Among them, the 2 hp motor and the dynamometer are connected through a torque sensor. The test bearing type is 6205-2RS deep groove ball bearings. The diameter of the pitch circle of the bearing is 39mm, the contact angle is zero, the diameter of the rolling element is 8mm, and the motor speed is 1797rpm. During the experiment, the experimental data were measured by a vibration acceleration sensor mounted on the inner-ring of the drive end bearing. The sampling frequency was 12kHz and the inner-race fault size of the drive end bearing was 0.007-in.

#### Feature extraction of inner-ring fault bearing

**Fig 9:**
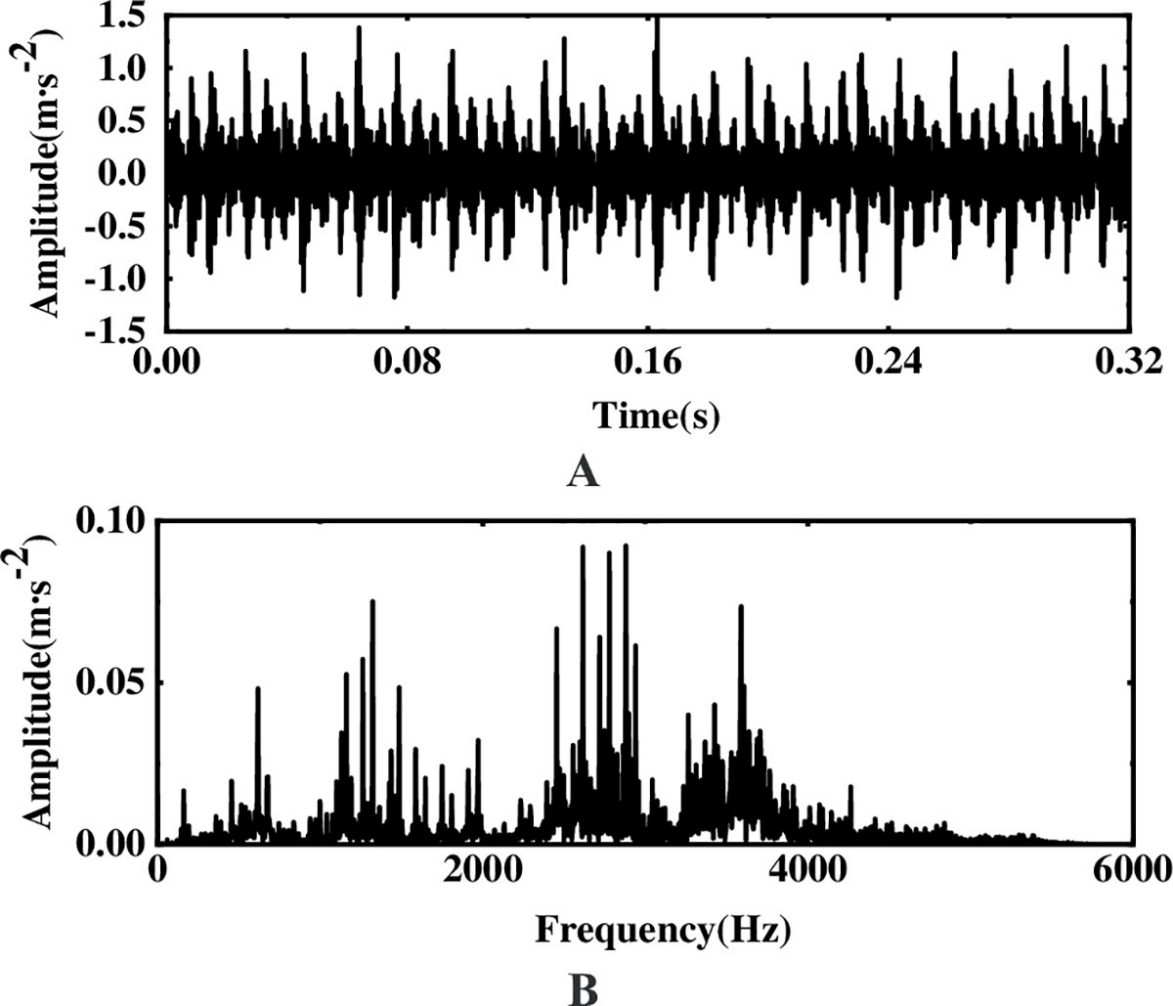
Time-domain waveform and spectrogram of inner-race fault. (A) Original waveform (B) Spectrogram.

In actual operation, due to the harsh working environment, multi-source vibration excitation, and the influence of human factors, the early vibration signal is weak and the fault characteristics are difficult to extract. This section studies the effectiveness of the proposed method under two different working conditions, working condition 1: motor speed 1797rpm, motor load 0hp; working condition 2: motor speed 1772rpm, motor load 1hp. The time-domain and frequency-domain diagrams of the vibration signal of the inner-ring fault bearing obtained under the first working condition are shown in Fig 9.

Although periodic shocks can be clearly seen in the time-domain waveform of Fig 9A, it is difficult to find fault characteristics from the waveform and FFT spectrum of Fig 9B due to the influence of external interference factors.

**Fig 10:**
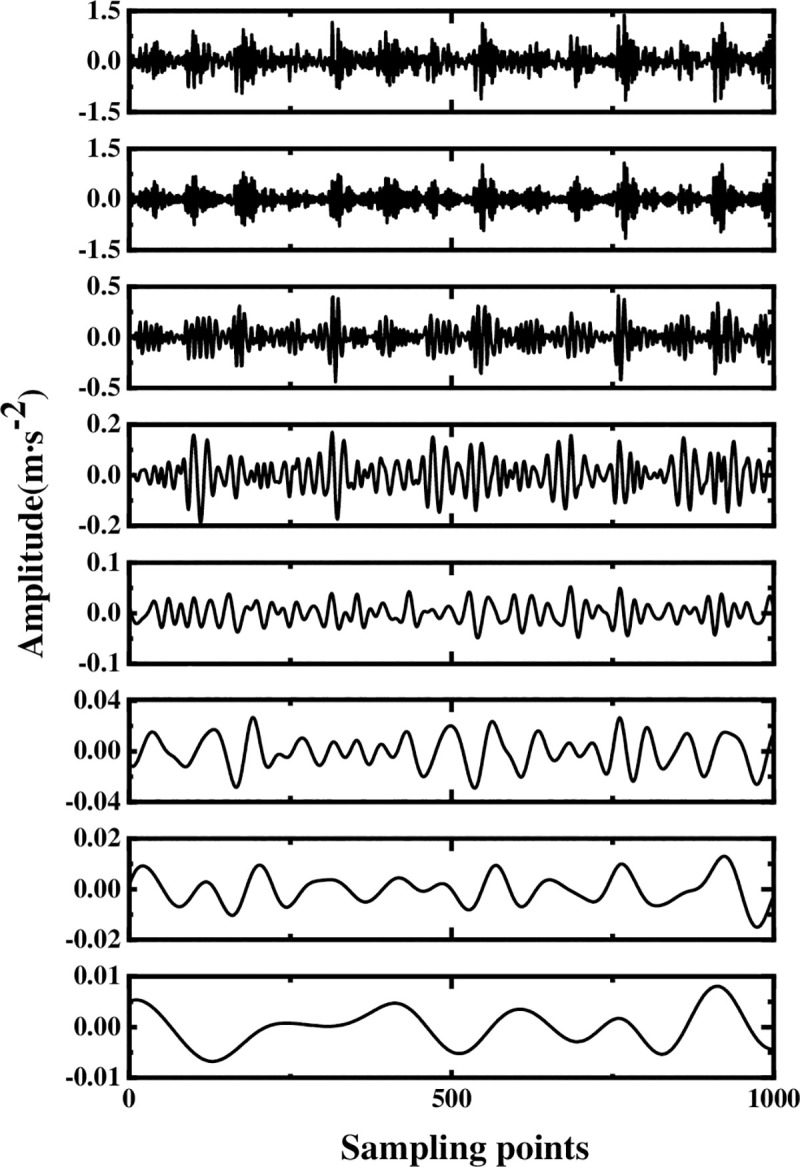
EEMD decomposition of vibration signal of inner-ring fault bearing.

**Fig 11:**
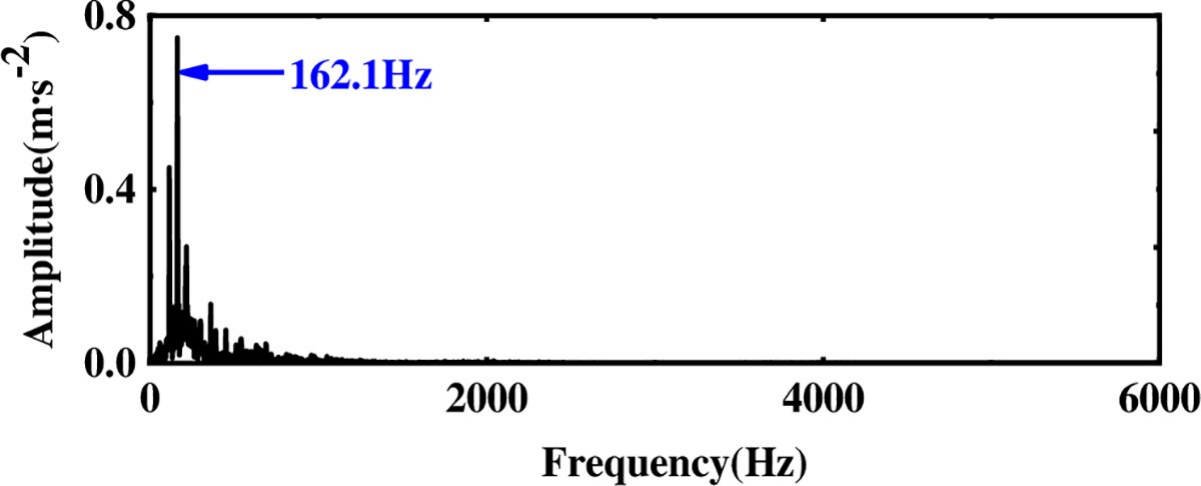
Spectrum diagram of vibration signal after multi-layer noise reduction.

The multi-layer noise reduction method proposed first needs to perform EEMD decomposition of the original signal to obtain IMF components. Considering that there are less useful signals in the low-frequency IMF components, only the first eight-order IMF components are drawn, as shown in Fig 10. Observing Fig 10, to select the main IMF components more accurately, the comprehensive evaluation index is used to select the components occupying more than 85% of the original signal for analysis. According to Table 2, it is found that the first three orders of IMF components contain more than 85% of the information of the original signal. Therefore, the noise reduction process is performed on the first three orders of the IMF component. Simulation analysis shows that WTD has a certain noise reduction effect on the signal, but it is not very effective for feature extraction. Therefore, the first three-order IMF components are used as the observation signal after WTD noise reduction, the remaining components are summed as the virtual noise signal, and the FastICA algorithm is used for noise reduction and unmixing. Fig 11 shows the frequency spectrum of the proposed method to process the original vibration signal and it is observed that the amplitude at 162.1 Hz is obvious. Comparing the frequency spectrum of the original vibration signal, it is found that after the vibration signal of the inner-ring fault has been processed by multi-layer noise reduction, the characteristic signal is displayed; when the motor speed is 1797rpm, the fault frequency of the rolling bearing is calculated to be 162.1Hz according to the bearing specification. The theoretical calculation value is consistent with the experimental analysis of the fault frequency, which again verifies the effectiveness in extracting weak characteristic signals of rotating machinery.

**Table 2:** The *Q*_*i*_ value of IMF component of the vibration signal.

	IMF_1_	IMF_2_	IMF_3_	IMF_4_	IMF_5_	IMF_6_
H_1_	0.573	0.212	0.105	0.062	0.023	0.012
H_2_	0.258	0.223	0.198	0.132	0.119	0.064
H_3_	0.624	0.251	0.067	0.021	0.012	0.011
H_4_	0.702	0.193	0.042	0.031	0.024	0.003
Q_i_	0.539	0.220	0.103	0.062	0.045	0.023

**Fig 12:**
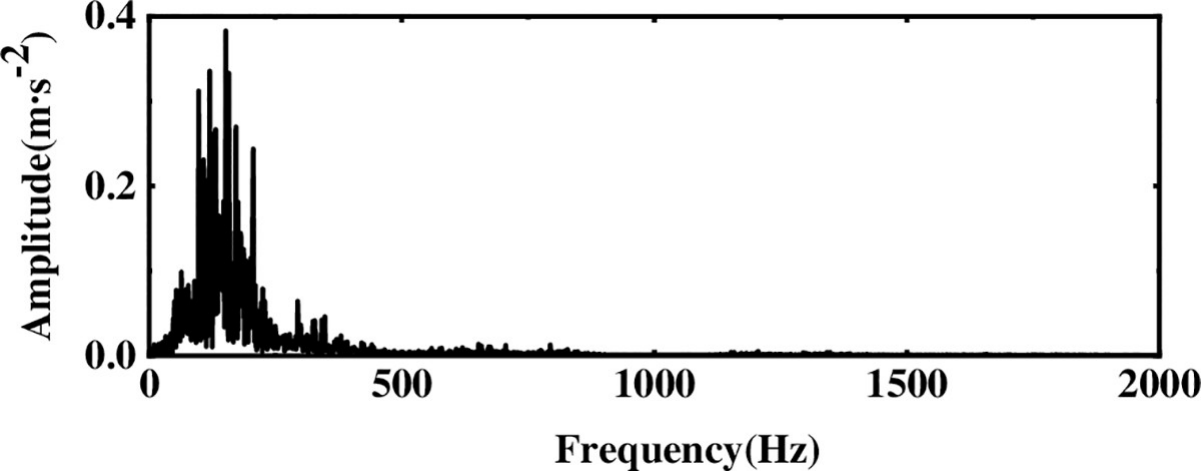
Spectrogram after EEMD-WTD-SVD processing.

Since WTD has a good effect on noise suppression, it is not very effective in extracting weak signal features. To verify the advantages of ICA in extracting features in the proposed method, the EEMD-WTD-SVD method is used to process the original signal as shown in Fig 12. Comparing Figs 11 and 12, it is further verified that ICA has certain advantages for the extraction of weak characteristic signals while reducing noise interference. The results show that the effectiveness of the method proposed is not only achieved by WTD, but by EEMD decomposition, selection of IMF components, WTD and ICA.

**Fig 13:**
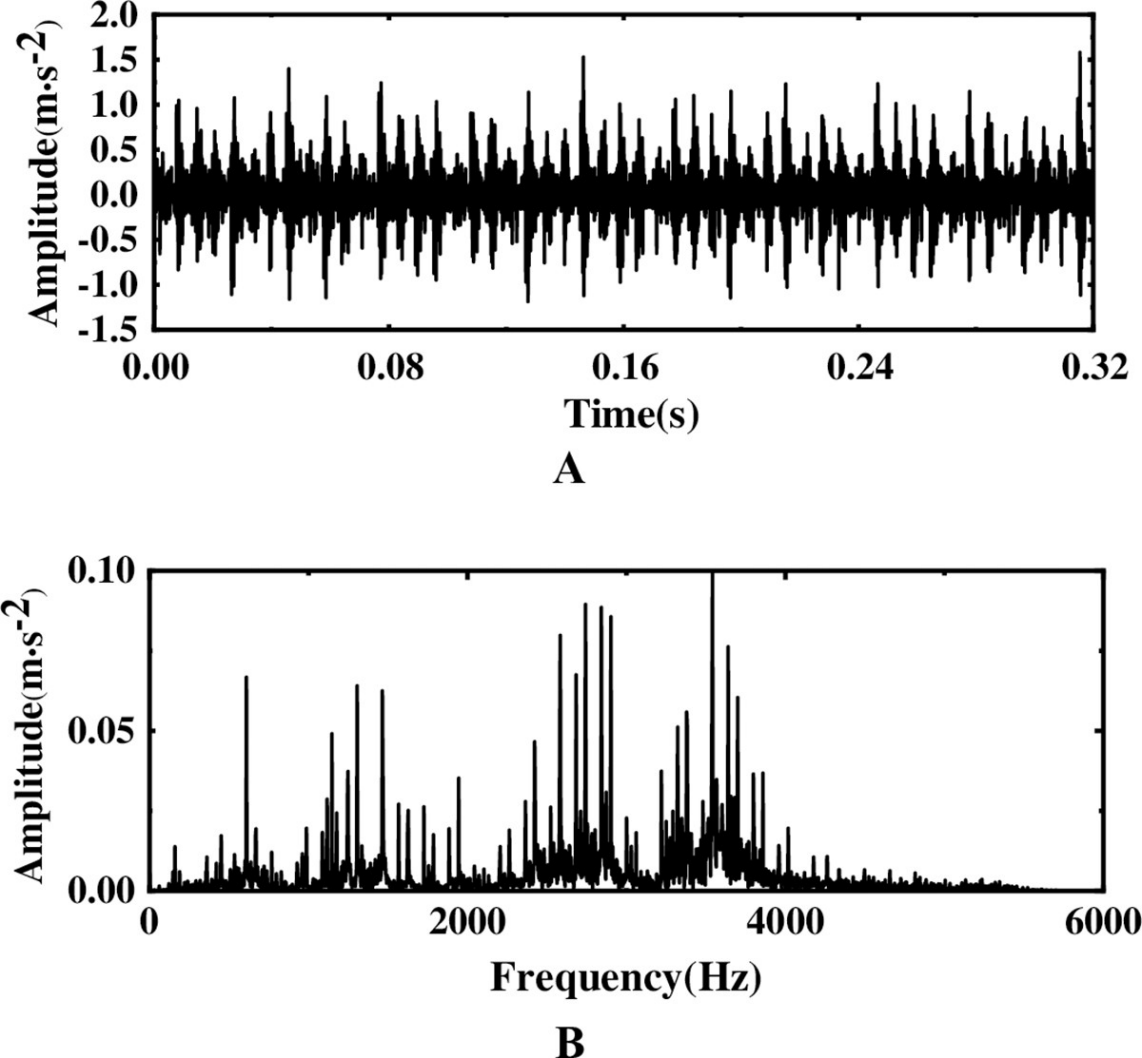
Time-domain and frequency-domain diagrams of the original signal under working condition 2. (A) Time-domain graph (B) Frequency domain graph.

**Fig 14:**
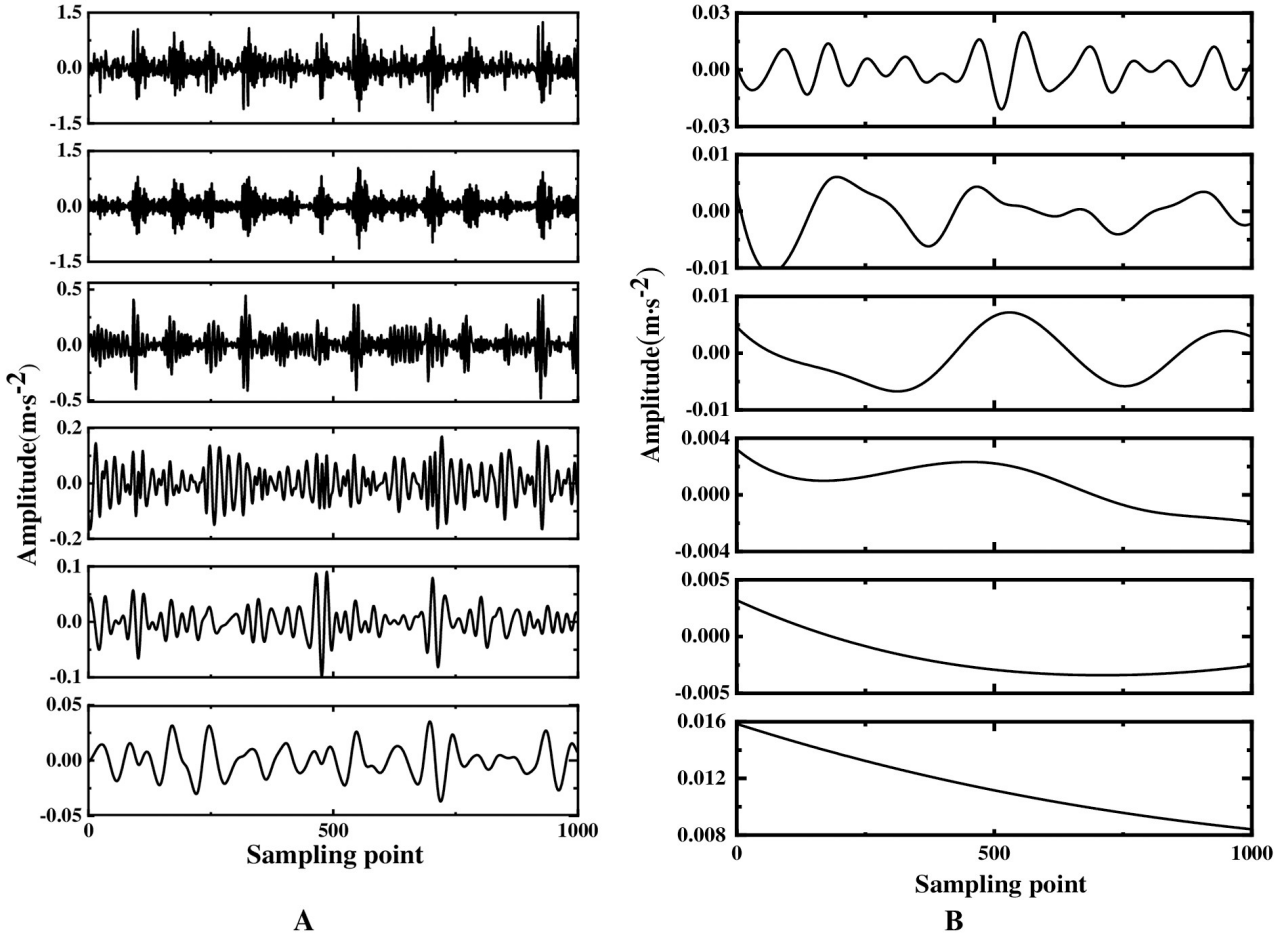
IMF components after EEMD decomposition of the original signal. (A) IMF1-IMF6, (B) IMF7-IMF11 and a residual component.

**Fig 15:**
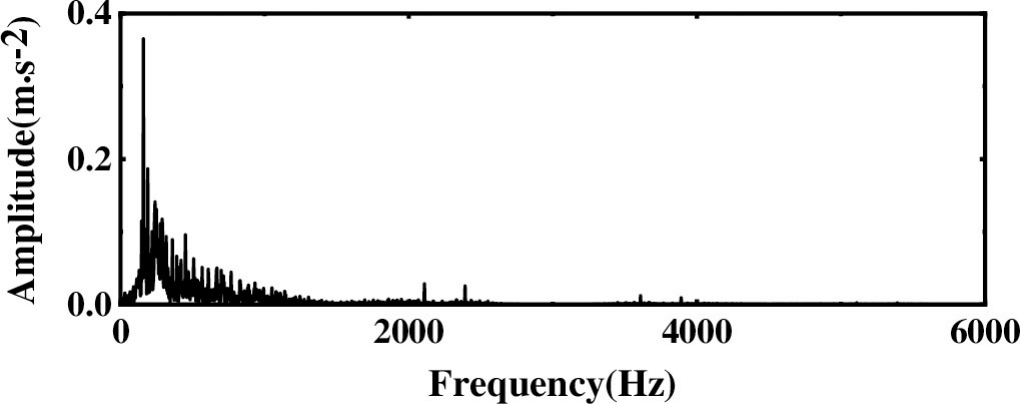
Spectrum diagram of the original signal after multi-layer denoising.

In addition, in order to verify the versatility of the proposed method, the vibration signal of the bearing under the second working condition is analyzed. First, obtain the time domain and FFT spectrograms of the bearing under working condition 2 as shown in Fig 13. The periodic signal in the time-domain waveform in Fig 13A indicates that the bearing has failed, but the fault frequency in Fig 13B is difficult to highlight due to the presence of external interference signals. For this reason, the multi-layer noise reduction method proposed in this paper is used to process the original signal. The IMF component of the original signal was obtained by EEMD decomposition. As can be seen from Fig 14, a total of 12 order was obtained. Then, the comprehensive evaluation index was used to select the main IMF component, as shown in Table 3. Since the former 4 order of the IMF component occupied more than 85% of the information of the original signal. Therefore, the former 4 order component was processed by WTD as the observation signal, the remaining components were summed as the virtual noise signal, and the two were input into FastICA for noise reduction and de-mixing. The spectrum diagram after processing is shown in Fig 15. By comparing Fig 13B with Fig 15, it is found that the proposed multi-layer noise reduction method can effectively reduce the noise and highlight the characteristic frequency. Through the above two experiments, the results show that the proposed method can extract the fault characteristics of rotating machinery under different working conditions.

**Table 3:** The *Q*_*i*_ value of IMF component of the vibration signal.

	IMF_1_	IMF_2_	IMF_3_	IMF_4_	IMF_5_	IMF_6_
H_1_	0.561	0.231	0.126	0.054	0.014	0.006
H_2_	0.229	0.176	0.152	0.184	0.106	0.134
H_3_	0.642	0.196	0.083	0.042	0.015	0.009
H_4_	0.763	0.122	0.059	0.026	0.014	0.004
Q_i_	0.548	0.181	0.105	0.076	0.037	0.038

### Engineering application

#### Data acquisition

**Fig 16:**
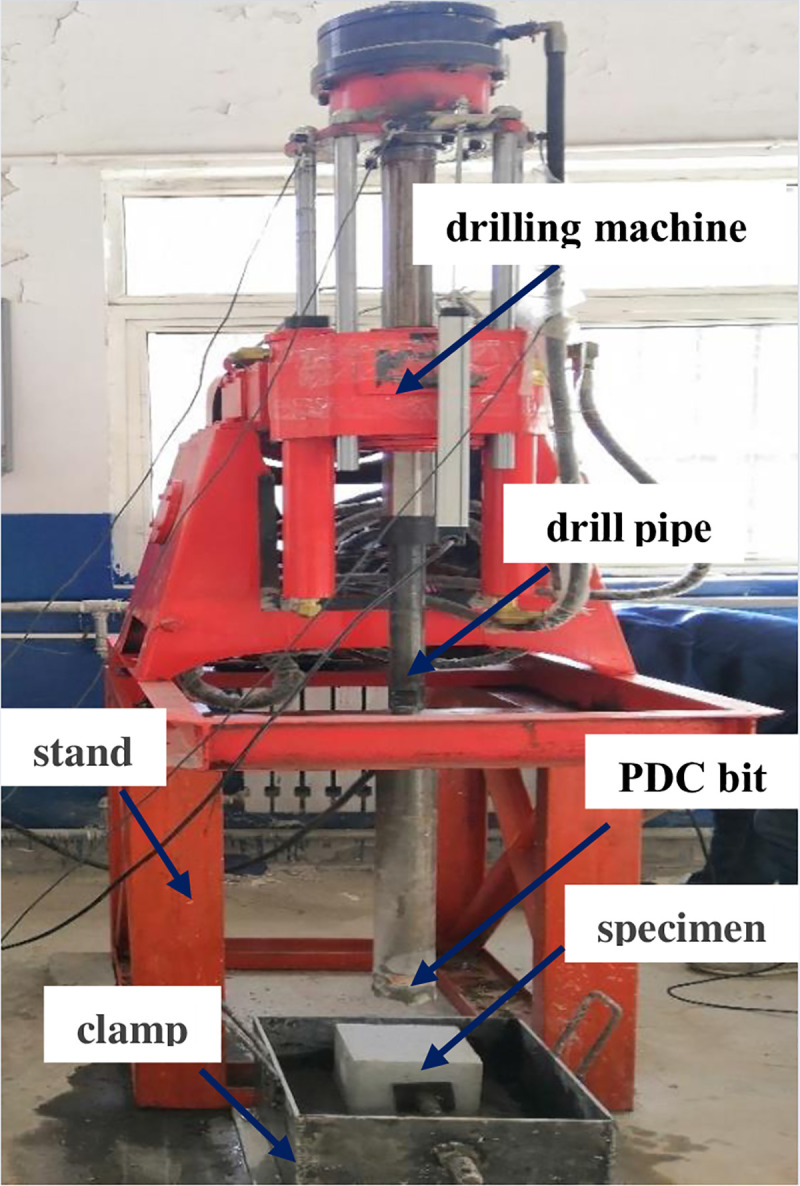
Experimental drilling machine.

**Fig 17:**
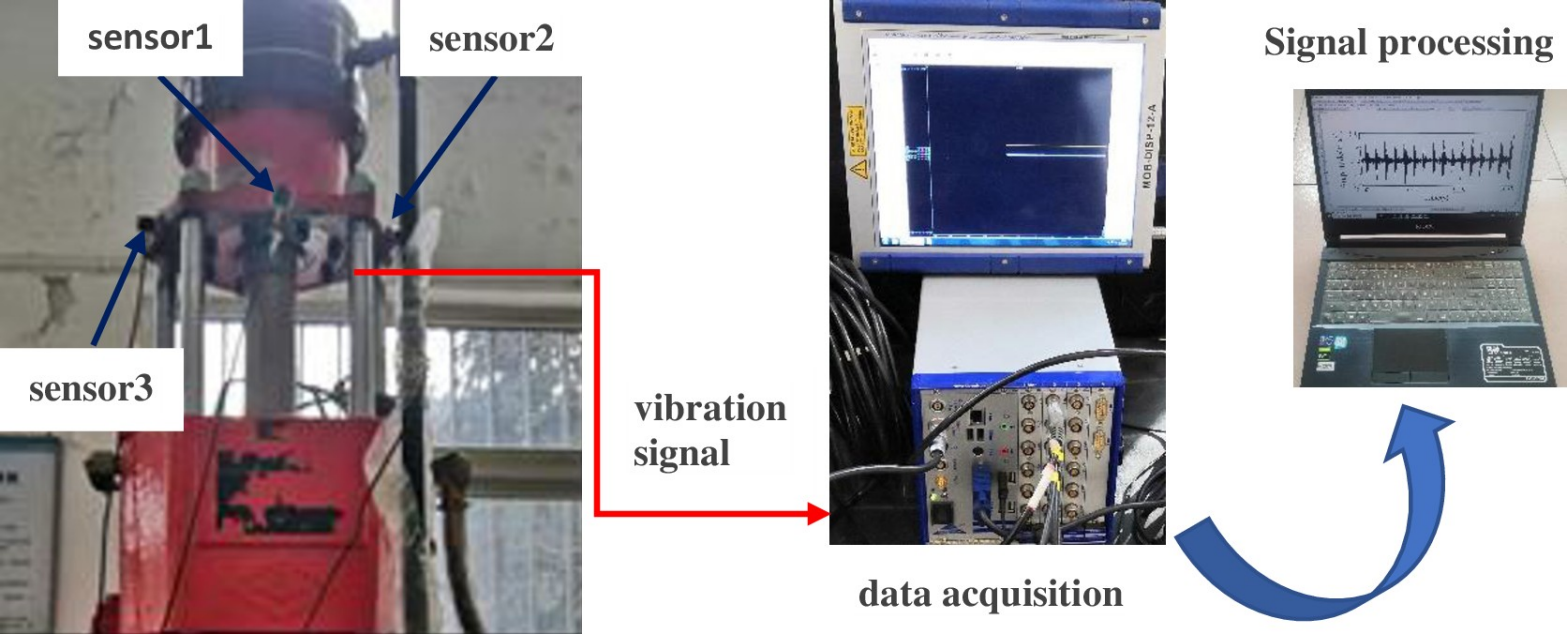
Sensor placement and data processing.

The above experimental studies prove that the multi-layer noise reduction method proposed in this paper can carry out feature extraction for fault signals under different working conditions. Thus, in this section, to verify the practicability of the proposed method, the method proposed is applied to the extraction of weak signals in the working process of worn core bits. Among them, the drilling machine uses the ZLJ-350 tunnel drilling rig for coal mines. The drilling machine is connected to the ground through a stand. The experimental device is shown in Fig 16, which includes a drill pipe and a clamp for holding the specimen. The specimen is a concrete block with a compressive strength of 40 MPa cast by cement. During the experiment, the vibration signal was obtained using the FA1105-A1 acceleration sensor. The layout of the sensor is shown in Fig 17. Considering that axial vibration mainly occurs in the process of drilling and coring, in the following analysis, the data measured by sensor 1 is taken as the analysis object. The vibration signal is collected and analyzed by DEWESoft v6.6, the sampling frequency is set to 20kHz, the drilling speed of the drill bit is 110rpm, and the inner diameter of the core bit is 52mm. In the process of data collection, due to the influence of the vibration signal transmission path and multi-source vibration excitation, the obtained signal has a large amount of interference noise. Therefore, the proposed method is used to extract the feature of the weak signal.

#### Feature extraction of worn PDC bit

**Fig 18:**
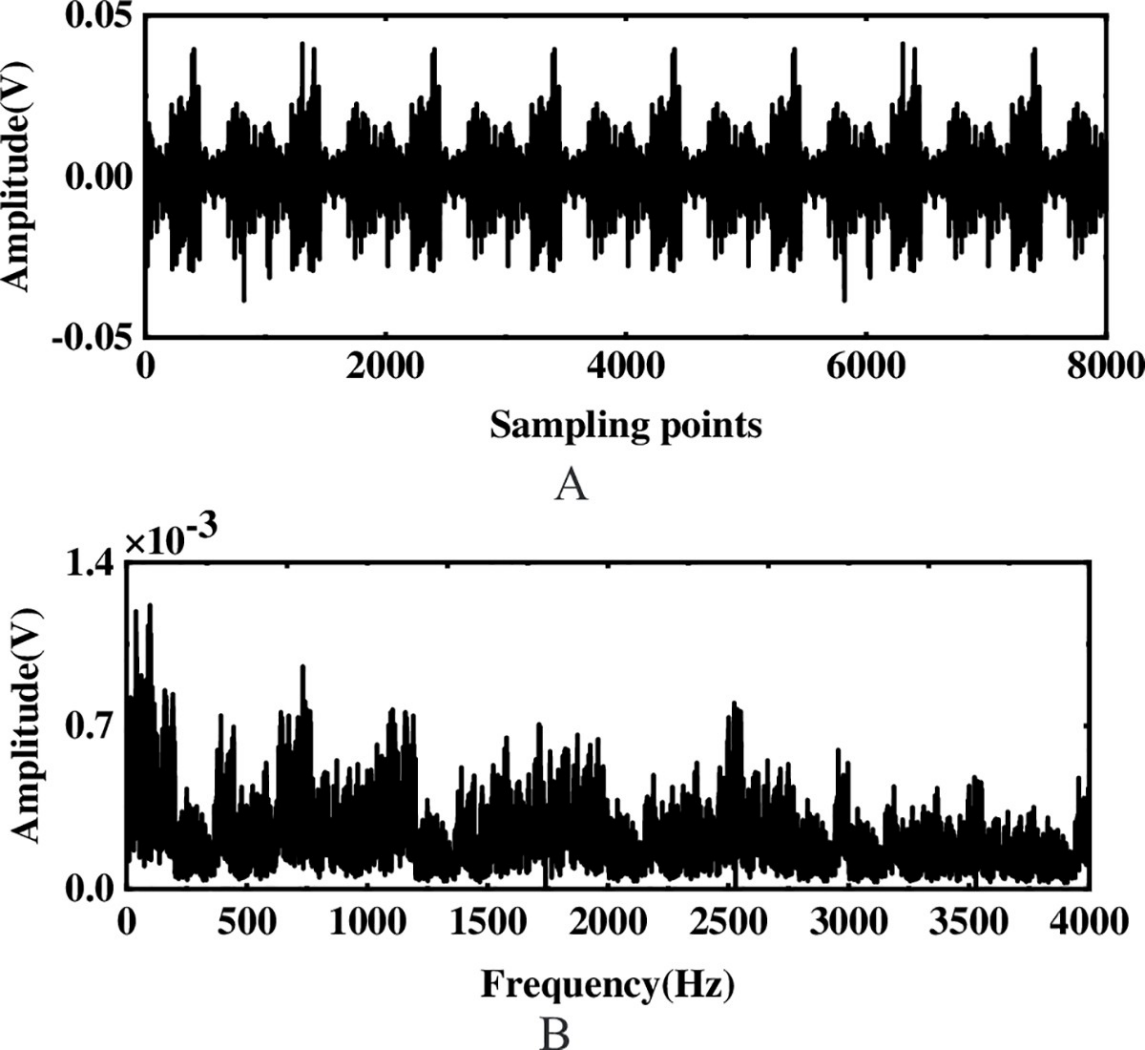
Time-domain frequency domain diagram of worn PDC bit vibration signal. (A) Original time domain (B) Frequency domain diagram.

**Fig 19:**
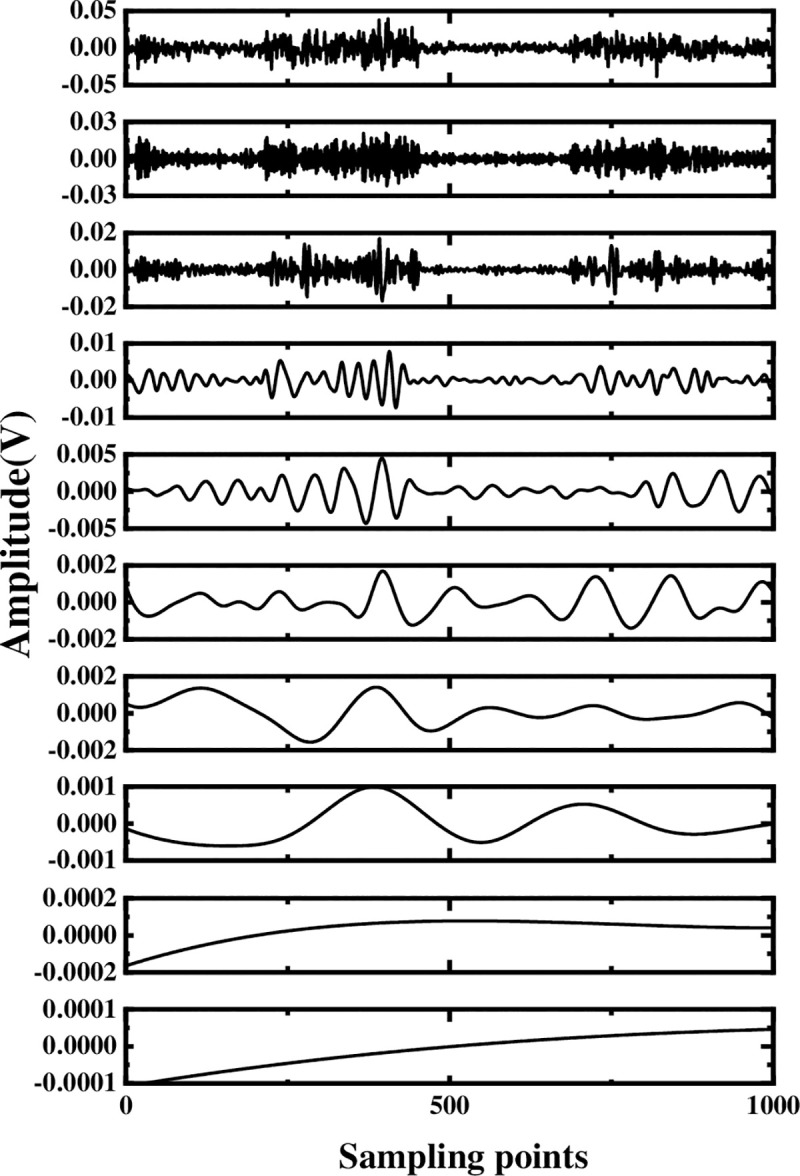
Vibration signal EEMD decomposition IMF components.

**Fig 20:**
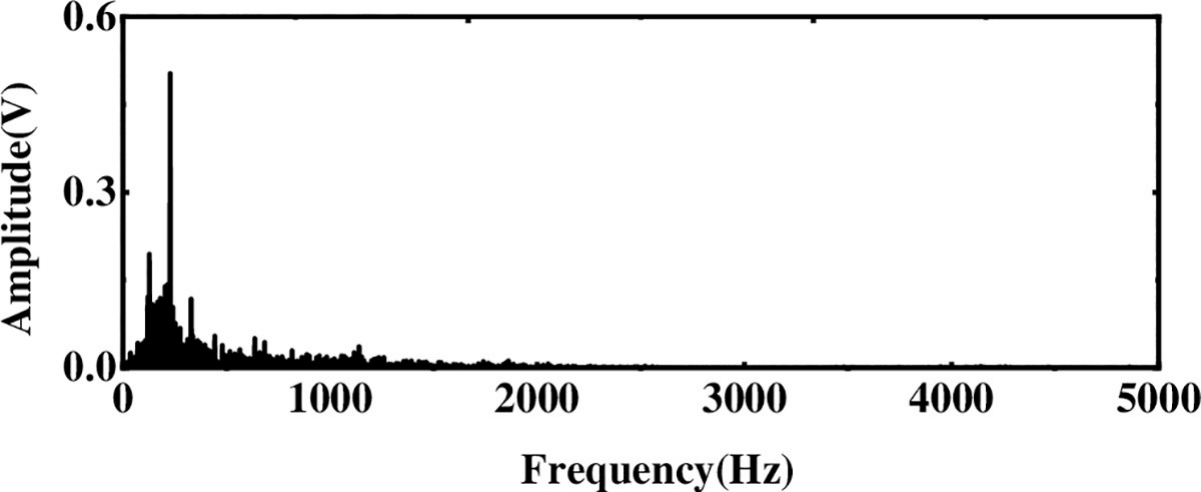
The frequency spectrum of the vibration signal after multi-layer noise reduction.

Considering that the characteristic frequency of the worn bit vibration signal will appear periodically in a short time, observe the original waveform of the worn bit and the FFT spectrum graph as shown in Fig 18. Although obvious periodic shock signals can be observed in the time domain diagram, the characteristic frequencies in the spectrogram are difficult to highlight due to the influence of external noise and human factors during the vibration signal measurement process. Therefore, it is necessary to de-noise the vibration signal. The original signal is decomposed by EEMD to obtain IMF components, as shown in Fig 19. Due to the presence of noise, the IMF component contains false components, and as the frequency of the IMF component decreases, the less useful signal is contained. To reduce the noise interference, the comprehensive evaluation index of the first six-order IMF components is calculated, and the IMF components with more original signals are selected. Observing Table 4, we can see that the first four-order IMF components contain more than 85% of the original signal. Therefore, the first four-order IMF components are selected for further noise reduction processing. To highlight the characteristic signals, FastICA analysis is performed on the IMF components. Among them, the first four-order IMF components are subjected to WTD noise reduction and used as the observation signal, and the remaining IMF components are summed as the virtual noise signal. By taking the observation signal and the virtual noise signal as the input matrix of the ICA, and using FastICA for noise reduction and unmixing, the final spectrogram of the vibration signal after noise reduction is obtained, as shown in Fig 20.

**Table 4:** Q_i_ value of vibration signal IMF.

	IMF_1_	IMF_2_	IMF_3_	IMF_4_	IMF_5_	IMF_6_
H_1_	0.552	0.243	0.102	0.066	0.021	0.005
H_2_	0.216	0.173	0.155	0.162	0.149	0.132
H_3_	0.536	0.167	0.125	0.091	0.052	0.013
H_4_	0.526	0.228	0.156	0.043	0.006	0.003
Q_i_	0.458	0.203	0.135	0.091	0.057	0.038

**Fig 21:**
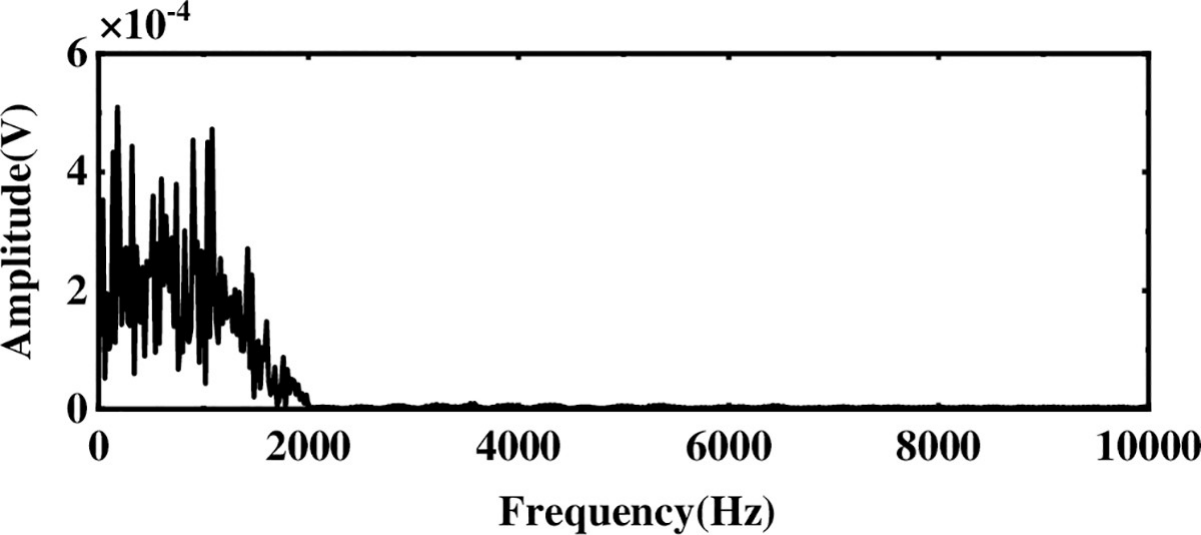
The frequency spectrum of LMD-WTD processing vibration signal.

**Fig 22:**
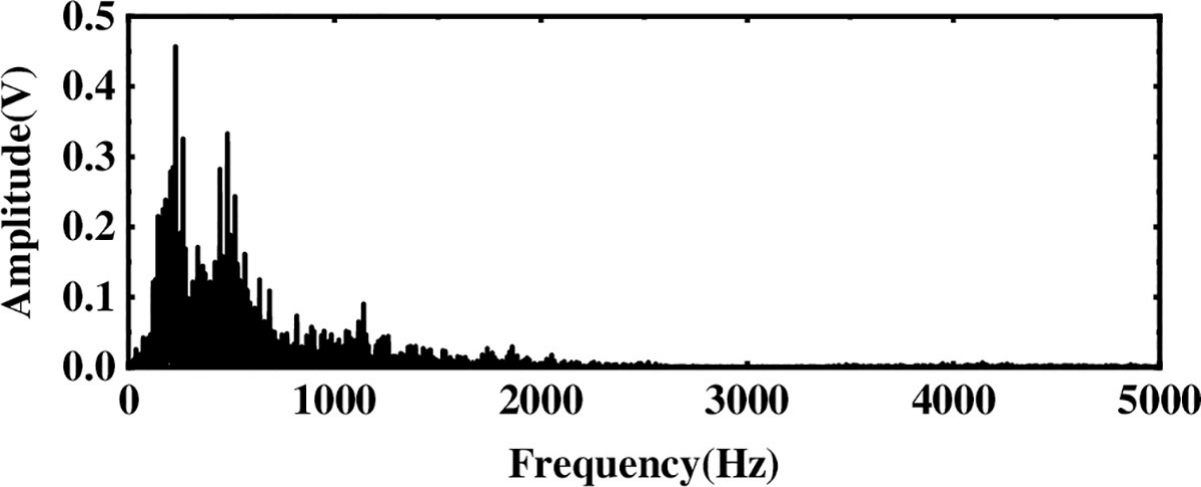
The frequency spectrum of EEMD-ICA processing vibration signal.

To verify the effectiveness of the method proposed in this paper, using LMD-WTD [25] and EEMD-ICA [40] to process the frequency spectrum of vibration signals are shown in Figs 21 and 22. Observing Fig 21, it is obvious that WTD can suppress noise to a certain extent, but WTD is not very effective for extracting weak signals. In Fig 22, although the characteristics of the weak signal can be highlighted to a certain extent, the noise suppression effect when using EEMD-ICA to extract the weak signal is not very good; comparing Figs 20–22, it verifies the noise suppression effect of WTD and the advantage of ICA in weak signal extraction.

## Conclusions

Aiming at the problem of extracting weak fault features in strong noise, this paper proposes a multi-layer noise reduction method combining comprehensive evaluation indicators, WTD and ICA. Experimental analysis and engineering applications have verified the practicability and effectiveness of the method. The conclusions are as follows:

1. The use of EEMD to decompose the original signal solves the problem of the loss of characteristic information caused by the modal aliasing and end effect of EMD. Using EEMD decomposition to obtain a series of IMF components, blindly choosing IMF components may cause the loss of fault characteristics. For this reason, this paper proposes a comprehensive evaluation index to select the main IMF components.
2. To extract the characteristic frequency of weak signals, considering that WTD has a certain inhibitory effect on high-frequency noise, WTD is used as the pre-filter of ICA to process the main IMF components.
3. The main IMF components processed by WTD are used as observation signals, and the remaining IMF components are input into ICA as virtual noise channel signals; the introduction of virtual channels effectively solves the problem of underdetermined blind source separation in the ICA algorithm. Experimental analysis shows that the proposed multi-layer noise reduction method is obvious for the extraction of weak signals under complex working conditions in engineering applications, and it is theoretically suitable for weak signal detection of various types of rotating machinery.
